# Impact of Conventional and Laser-Assisted Machining on the Microstructure and Mechanical Properties of Ti-Nb-Cr-V-Ni High-Entropy Alloy Fabricated with Directed Energy Deposition

**DOI:** 10.3390/mi15121457

**Published:** 2024-11-29

**Authors:** Ho-In Jeong, Osama Salem, Dong-Won Jung, Choon-Man Lee, Jeung-Hoon Lee

**Affiliations:** 1 Mechatronics Research Center, Changwon National University, Changwon 51140, Gyeongsangnam-do, Republic of Korea; jhi8443@changwon.ac.kr; 2 Department of Production Engineering and Mechanical Design, Faculty of Engineering, Menoufia University, Shebin El-Kom 32511, Egypt; osama.mohammed@sh-eng.menofia.edu.eg; 3 Faculty of Applied Energy System, Major of Mechanical Engineering, Jeju National University, Jeju-si 63243, Jeju-do, Republic of Korea; 4 Department of Smart Manufacturing Engineering, Changwon National University, 20, Changwon-daehak-ro, Uichang-gu, Changwon-si 51140, Gyeongsangnam-do, Republic of Korea; jhoonlee@changwon.ac.kr

**Keywords:** additive manufacturing, Directed Energy Deposition, high-entropy alloy, laser-assisted machining, post-processing

## Abstract

The high-entropy alloy (HEA) has recently attracted significant interest due to its novel alloy design concept and exceptional mechanical properties, which may exhibit either a single or multi-phase structure. Specifically, refractory high-entropy alloys (RHEA) composed of titanium, niobium, and nickel-based HEA demonstrate remarkable mechanical properties at elevated temperatures. Additive manufacturing (AM), specifically Direct Energy Deposition (DED), is efficient in fabricating high-entropy alloys (HEA) owing to its fast-cooling rates, which promote uniform microstructures and reduce defects. This study involved the fabrication of the Ti33Nb28Cr11V11Ni17 (Ti-Nb-Cr-V-Ni) RHEA utilizing DED. Additionally, the post-processing of the fabricated alloy is conducted using conventional machining (CM) and laser-assisted machining (LAM). The results indicate thermal conductivity and specific heat increased, whereas tensile strength reduced with rising temperature. Significant softening was observed above 800 °C, resulting in a considerable decrease in tensile strength. Furthermore, the LAM caused material softening and reduced the cutting force by 60.0% relative to CM. Furthermore, the chemical composition of Ti-Nb-Cr-V-Ni remained unaffected even after post-processing with CM and LAM. The research indicates that post-processing with LAM is essential for developing resilient RHEA for practical use.

## 1. Introduction

High-entropy alloy (HEA) has garnered significant attention due to its innovative alloy design concept and remarkable mechanical properties as an advanced concept alloy. HEAs comprise 5 to 35 at% of five or more elements with comparable atomic radius and exhibit high configurational entropy, lattice distortion, and slow diffusion rate [1]. Owing to these characteristics, HEA does not exhibit the formation of intermetallic compounds and tends to form single-phase solid solutions [2]. Due to their distinctive composition and microstructure, HEA has exceptional mechanical properties.

Particularly, refractory high-entropy alloys (RHEA), comprise refractory materials such as titanium, niobium, vanadium, molybdenum, chromium, tantalum, and tungsten and nickel-based HEA comprise FCC single-phase solid solutions exhibit outstanding mechanical characteristics at elevated temperatures. These properties are comparable to those of titanium and nickel-based alloys, which are extensively employed in extreme environments due to superior heat resistance and specific strength [3,4]. However, most of the investigations primarily concentrate on the addition of titanium and nickel, thus precluding their usage as primary constituents in HEA. Consequently, no research has been conducted on HEA comprising titanium and nickel, which exhibit excellent properties. In this study, the HEA comprised Ti-Nb-Cr-V-Ni was manufactured through Directed Energy Deposition (DED), a representative additive manufacturing (AM) method. DED is an AM technique that employs focused thermal energy to melt simultaneously and deposit materials [5]. DED is advantageous for grain refining because of its rapid deposition rate, rapid cooling, and swift solidification, and can mitigate. Due to these characteristics, many researchers are currently conducting research on the manufacture of HEA using DED [6].

Most of the materials manufactured using DED exhibit the disadvantages of having highly irregular surface quality and uncontrolled surface roughness [7]. Since most types of mechanical failure are susceptible to surface properties, improving the surface quality, which affects the performance and lifetime of DED materials, is an important issue [8]. Numerous post-processing techniques have been investigated to improve the properties of high-entropy alloys (HEAs) produced via additive manufacturing. Methods include hot isostatic pressing (HIP), which reduces porosity and enhances density; traditional heat treatments (HTs), which optimize microstructure and mechanical properties; and surface modification techniques such as laser-based treatments, shot peening, and electrochemical polishing. Each method is essential for refining microstructure, enhancing surface quality, and improving the mechanical performance of HEAs.

Laser-assisted machining (LAM) enhances surface finishes and reduces thermal distortion, proving especially effective for challenging materials such as high-entropy alloys (HEAs). Utilizing these post-treatment techniques, researchers seek to maximize the potential of HEAs for diverse high-performance applications [9]. Machining is the most used post-processing method due to its high efficiency and low cost, and research on hybrid additive manufacturing (HAM) combining the DED process and machining is being actively pursued [10].

However, titanium and nickel-based alloys and HEA are categorized as difficult-to-cut materials because of their high mechanical characteristics including high hardness, strength and work hardening, and low thermal conductivity [11]. The difficult-to-cut material poses challenges for conventional machining (CM) due to their low machinability and consequently, low productivity stemming from excessive tool wear and high cutting force [12]. To solve this problem, thermally assisted machining (TAM) emerged as a solution for machines to difficult-to-cut material effectively by utilizing auxiliary heat sources including laser, induction, and plasma. TEM is a processing method that softens the workpiece using external heat sources to reduce its strength for efficient machining [13]. In machining ductile materials, brittle fracture is suppressed, and cutting force is reduced, enabling efficient machining [14]. Laser-assisted machining (LAM) uses a laser with high energy density as a heat source to quickly soften the material, and the heat-affected zone (HAZ) is narrow, with no thermal effect including thermal deformation outside the machining area [15].

Zhai et al. [16] used LAM to machine C/SiC composite material and verified the efficiency of LAM. Anderson et al. [17] Confirmed the 3-D heat transfer model for the LAM and estimated the machining characteristics for the LAM of Inconel 718. Feng et al. [18] used LAM for the machining of Inconel 718 and developed an analysis model for roughness prediction. The analysis model showed a minimum error of 3%, and the surface quality increased in LAM. Wang et al. [19] used LAM for the machining of Al_2_O_3_p/Al composite material and compared cutting force, surface roughness, and tool wear of CM and LAM to verify the efficiency of LAM. Woo et al. [20] applied LAM as a post-process of manufactured Ti-6Al-4V using DED and, verified LAM had improved machining efficiency than CM.

**Figure 1:**
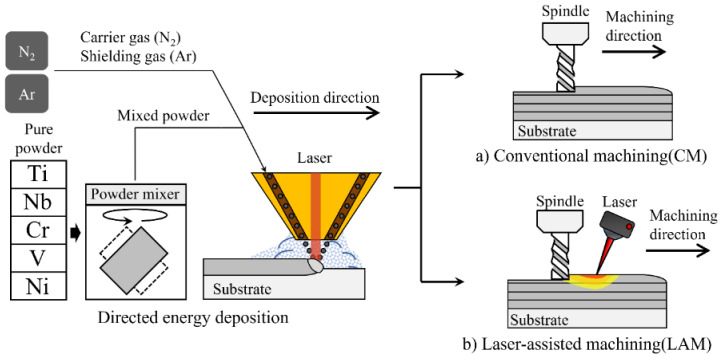
The schematic of DED and post-processing using (**a**) conventional machining (CM) and (**b**) laser-assisted machining (LAM).

Despite these studies, limited research work is carried out on the post-processing of HEA fabricated using AM. Furthermore, studies using LAM as the post-processing of HEA are minimal. Therefore, this study evaluates the material properties of deposited Ti-Nb-Cr-V-Ni using DED, and the machining effect and characteristics are analyzed by applying CM and LAM for post-processing. Figure 1 shows a schematic of DED and post-processing using CM and LAM. For the deposit of Ti-Nb-Cr-V-Ni, a phase diagram was analyzed to determine the DED conditions. And each powder was mixed to manufacture Ti-Nb-Cr-V-Ni using DED. For the evaluation of deposited Ti-Nb-Cr-V-Ni, an analysis of the microstructure, tensile strength, and thermal conductivity was conducted. To decide the cutting depth in post-processing through LAM, the Ti-Nb-Cr-V-Ni temperature distribution in response to the laser heat source was analyzed, and appropriate preheating temperature and preheating depth were analyzed. Finally, the efficiency of LAM was verified by analyzing and comparing machining characteristics including cutting force, surface roughness, microhardness, and microstructure resulting from CM and LAM.

## 2. Materials and DED Processing

**Figure 2:**
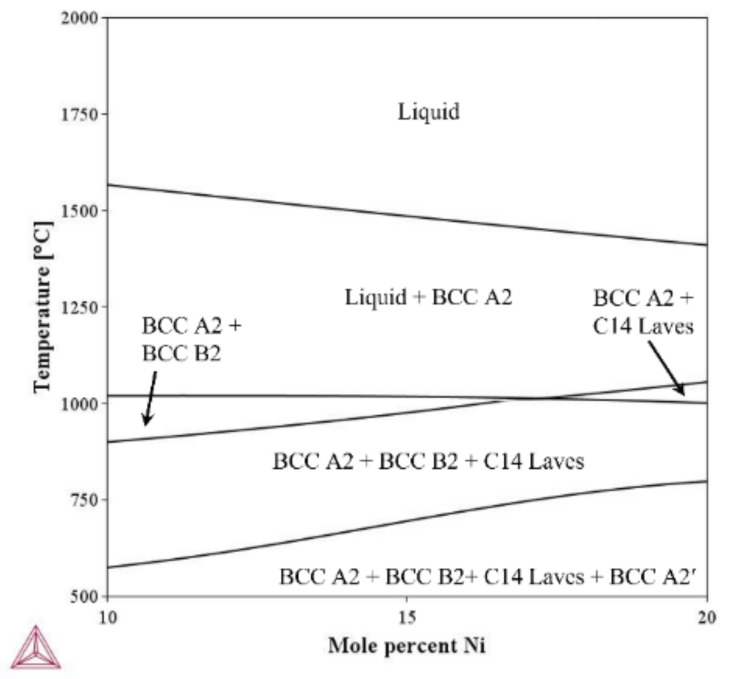
The phase diagram for Ti-Nb-Cr-V-Ni.

To decide the DED processing temperature a phase analysis of the developed alloy was conducted using Thermo-Calc software 2023b and the TCFE12 database under equilibrium conditions. Figure 2 shows the developed phase diagram for Ti-Nb-Cr-V-Ni. Above the melting point of 1451 °C, Ti-Nb-Cr-V-Ni transforms from the liquid state to the solidification for the BCC A2 phase (Ti-Nb rich) with decreasing temperature. During solidification, the developed BCC A2 phase ratio plunged according to the growing BCC B2 phase (Ti-Ni rich) at 1013 °C. Further cooling resulted in the precipitation of the C14 Laves phase (Cr-Nb rich) at 1010 °C and the BCC A2′ phase (Cr-V rich) at 750 °C. Consequently, the DED temperature was decided to be over 1100 °C for solidification of the single BCC A2 phase solid solution and suppress precipitation of the secondary phase. Table 1 lists the alloy composition for Ti-Nb-Cr-V-Ni.

The alloy composition for Ti-Nb-Cr-V-Ni is presented in Table 1, detailing the atomic percentages of each constituent element. The mixing ratio of the powders was established according to the phase diagram, ensuring that the proportions of each element were preserved as follows: Ti: 33.0 atomic percent, Nb: 28.0 atomic percent, Cr: 11.0 atomic percent, and V: 11.0 atomic percent. Each powder was meticulously weighed and blended in a powder mixer for four hours to obtain the specified ratios. The mixing process is crucial for ensuring homogeneity and uniform distribution of elements, which are vital for attaining the desired phase characteristics during the DED process. The selected ratios were determined through phase diagram analysis, forecasting phase stability across different temperatures and compositions. They adhere to these ratios to promote the formation of the desired BCC A2 phase while minimizing the presence of secondary phases during solidification.

**Figure 3:**
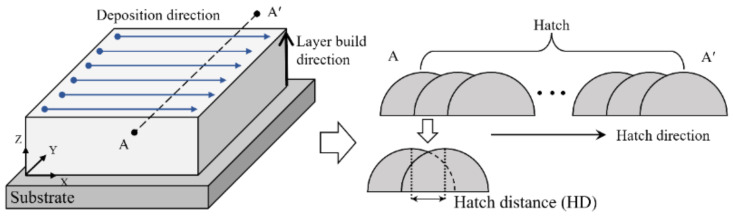

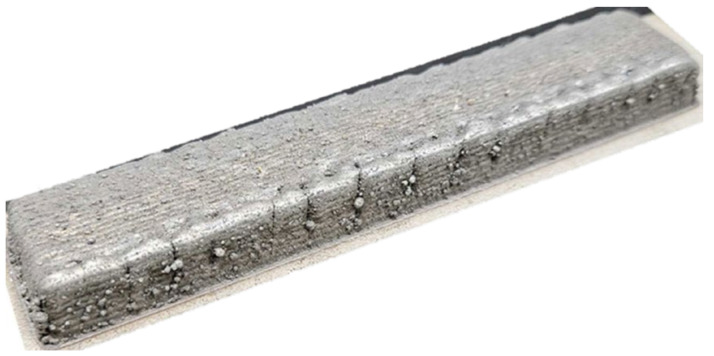
The schematic of the deposition path.

The Ti-Nb-Cr-V-Ni alloy was produced by blending powders with diameters ranging from 45 to 150 μm and a purity of 99.9%, subsequently deposited onto the Ti6Al4V substrate. The conditions for DED processing encompass laser power, scanning speed, powder feed rate, hatch distance, and shield gas flow rate. Figure 3 illustrates the schematics of the deposition path. The determination of hatch distance is critical for DED quality; an excessive hatch distance may lead to internal pores and cracks, adversely affecting mechanical properties, whereas a narrower hatch distance can result in an enlarged melt pool and a non-uniform bead formation, inducing internal stresses and decreasing production efficiency [21,22].

### 2.1. DED Equipment

**Figure 4:**
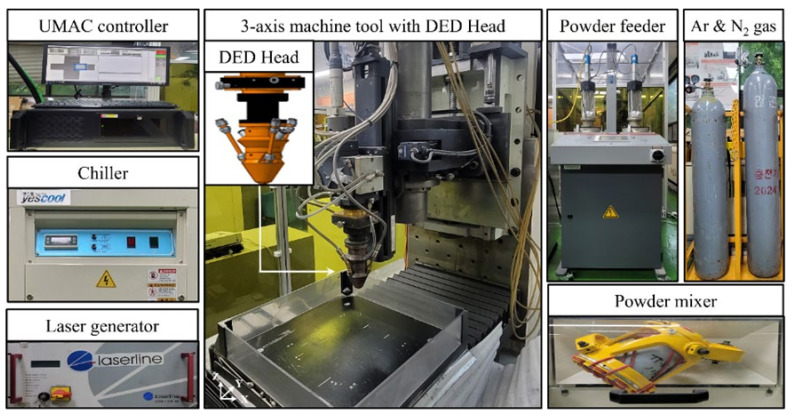
The DED equipment used to fabricate the HEA.

The DED equipment for the deposition of Ti-Nb-Cr-V-Ni was constructed with a machine tool and DED head as shown in Figure 4. The stroke of each axis of the machine tool was 500/500/300 mm, respectively (X/Y/Z). The DED head mounted on the machine tool was connected to the diode laser (LDM 2000, Laserline, Mülheim-Kärlich, Germany) with 2 kW power, 3 mm laser beam diameter, 198 mm focal length, and 980 nm wavelength. Additionally, a 780 W water chiller (YRC-1A, Yescool, Yongin City, Republic of Korea) for laser cooling was integrated into the DED equipment. To prepare the Ti-Nb-Cr-V-Ni powder, the powder mixer (MI-2T, DIM-NET, Machineweb, Inc., Deerfield, IL, USA) with 100 RPM rotational speed was used, and the Dual-pot volumetric powder feeder (Twin 150, Oerlikon Metco, Wohlen, Switzerland) transported the powder to the DED head. Nitrogen and argon gas serve as carrier gas and shielding gas for powder supply, respectively. DED system controls were managed by a Universal Machine and Automation Controller (UMAC), and the entire DED system was sealed to prevent oxidation caused by air ingress.

### 2.2. Experimental Procedure of LAM

CM and LAM post-processing of the deposited Ti-Nb-Cr-V-Ni was conducted to analyze the processing effect. The preheating temperature and feed rate by the laser heat source were set to 930 °C and 100 mm/min, respectively, based on the analysis of the thermally simulated process. The cutting depth was determined to be 0.3 mm, which is the depth at which the ductility of Ti-Nb-Cr-V-Ni increases significantly at 800 °C or higher. The spindle speed was selected under the conditions recommended for the machining of high-strength steel [23]. As a cutting tool, a flat-end mill (SM503080, Changwon-si, Republic of Korea) coated with TiAlN + SH was used to machine difficult-to-cut materials. Table 2 lists the machining conditions. The diode laser used at DED was mounted on the left side of the 5-axis machining center (Hi-V560M, Gwangmyeong-si, Republic of Korea) spindle.

**Figure 5:**
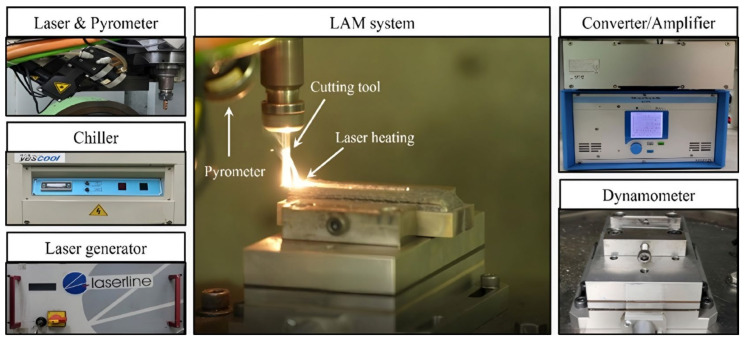
The LAM equipment.

Figure 5 shows the machines used for the LAM post-processing. To prevent damage to the cutting tool by the laser, the laser and cutting tool were positioned at a distance of 3 mm. Also, the same water chiller for cooling the laser, used for the DED was employed The pyrometer (LPC03, Dr. Mergenthaler GmbH and Co. KG, Neu-Ulm, Germany) capable of measuring from 400 to 3000 °C was mounted to the laser for measuring the surface temperature of the workpiece preheated by the laser. A dynamometer (9257B, KISTLER, Winterthur, Switzerland) was mounted to the 5-axis machining center to measure the cutting force. The cutting force on machining underwent amplification and conversion via an amplifier (5070, KISTLER), with cutting force subsequently transmitted to the PC utilizing the data acquisition board module (5697, KISTLER).

## 3. Results

### 3.1. Microstructural Analysis

To analyze the Ti-Nb-Cr-V-Ni microstructure, X-ray diffractometer (XRD) analysis, electron backscatter diffraction (EBSD) analysis, and energy dispersive spectrometer (EDS) analysis were used. According to the XRD and EBSD analysis, 88.83% of BCC A2 phase, 8.71% of BCC B2 phase, and 2.46% of Ti_2_Ni phase appeared in the microstructure of Ti-Nb-Cr-V-Ni, and the average grain size was 3.66 μm [24].

The Clarity EBSD detector from EDAX was employed for the EBSD analysis, functioning at an accelerating voltage of 20 kV. This configuration yielded high-resolution crystallographic data, facilitating the identification of grain orientations and the analysis of phase distributions within the microstructure. The data acquired from EBSD were essential for comprehending the textural characteristics of the alloy. The EDS analysis utilized the Octane SDD detector from EDAX, configured at an accelerating voltage of 20 kV. This configuration enabled precise elemental analysis, allowing for the quantification of the alloying elements in the Ti-Nb-Cr-V-Ni system. The integration of EDS and EBSD analyses yielded a thorough comprehension of the alloy’s microstructural and compositional attributes.

**Figure 6:**
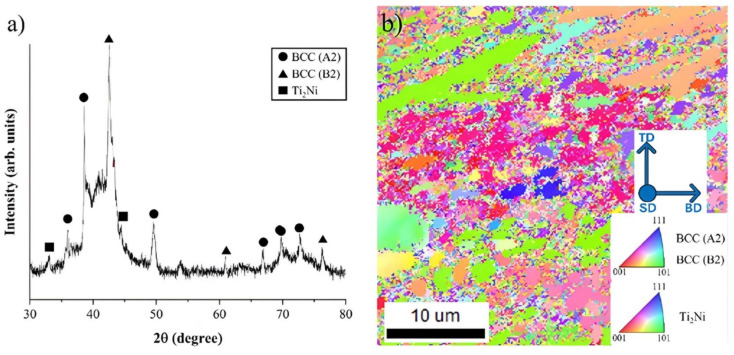
(**a**) XRD analysis and (**b**) The IPF map of Ti-Nb-Cr-V-Ni with the building direction (BD), scanning direction (SD), and transverse direction (TD).

The DED process utilizing a heat source with high energy density exhibits the heat treatment effect due to rapid temperature fluctuations during the process [25]. And Ti-Nb-Cr-V-Ni underwent rapid stabilization with high mixing configuration entropy appeared the fine grain size. Figure 6a shows the result of XRD and Figure 6b the Inverse Pole Figure (IPF) map of Ti-Nb-Cr-V-Ni.

**Figure 7:**
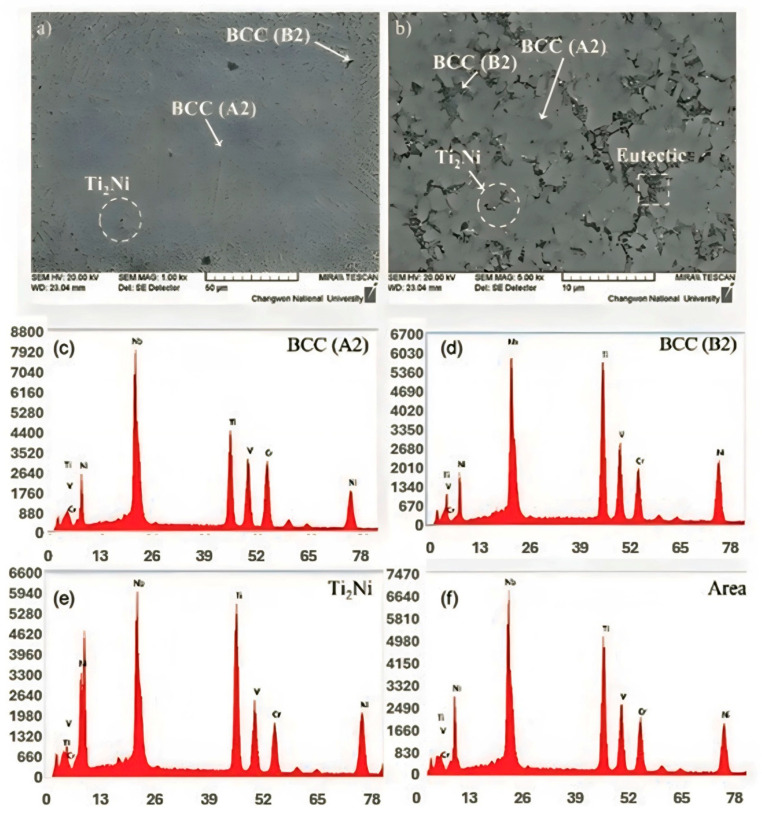
The SEM image of Ti-Nb-Cr-V-Ni (**a**) ×1000 magnifications, (**b**) ×5000 magnifications. EDS analysis with (**c**) BCC A2, (**d**) BCC B2, (**e**) Ti_2_Ni, and (**f**) total area.

Figure 7a,b present the SEM images of Ti-Nb-Cr-V-Ni with ×1000 and ×5000 magnifications, respectively. According to the SEM analysis result, the dendrite structure BCC A2 has grown significantly due to the rapid cooling. The BCC B2 phase was developed between BCC A2 phases, and the eutectic phase was formed to consist of BCC A2 and B2 phases arranged in the lamellar structure. The Ti_2_Ni precipitate was formed as the eutectic reaction of undissolved excess atoms during the solidification of the BCC B2 phase [26]. Figure 7c–f shows the EDS analysis of the developed HEA. According to the EDS analysis, the BCC A2 phase (Ti-Nb rich) was formed first, and then the BCC B2 phase (Ti-Ni rich) was formed. The Ti_2_Ni phase comprised undissolved Ti and Ni, so exhibited a Ti-Ni-rich composition as shown in Table 3. The composition of Ti-Nb-Cr-V-Ni was almost identical to the designed composition according to the EDS result, but there were subtle variations in each element’s concentration.

### 3.2. Mechanical Properties

**Figure 8:**
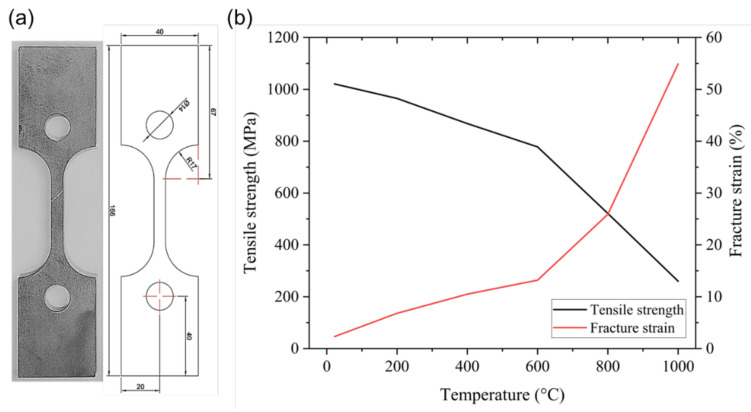
(**a**) the tensile test specimens, (**b**) The tensile strength and fracture strain of Ti-Nb-Cr-V-Ni according to temperature.

The tensile test was conducted to analyze the tensile strength of Ti-Nb-Cr-V-Ni at 22 to 1000 °C using a high-temperature tensile tester (M5582, Instron, Norwood, MA, USA). The specimens were manufactured to a thickness of 4 mm under the ASTM E8/E8M-18 standard. Figure 8a shows the tensile test specimens. The Ti-Nb-Cr-V-Ni exhibited high ultimate tensile strength but demonstrated brittle behavior at 22 °C. At 22 to 800 °C, it showed increased ductility, rapid softening occurred above 800 °C, and at 1000 °C the near-steady-state flow phase after yielding was prolonged. Figure 8b shows the tensile strength and fracture strain of Ti-Nb-Cr-V-Ni as a function of temperature. The high strength of Ti-Nb-Cr-V-Ni was analyzed as fine grain size by rapid cooling, a characteristic of the DED process, and solid solution strengthening of the solid solution formed by fast phase stabilization due to high mixing configurational entropy [27,28].

### 3.3. Thermal Conductivity

**Figure 9:**
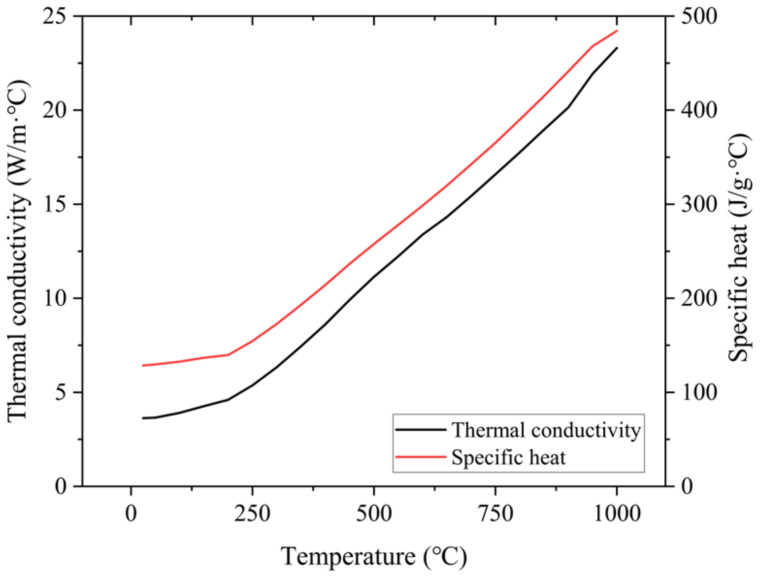
The thermal conductivity and specific heat of Ti-Nb-Cr-V-Ni according to temperature.

To analyze the thermal conductivity and specific heat of the Ti-Nb-Cr-V-Ni, the laser flash apparatus (LFA 457, NETZSCH, Selb, Germany) and differential scanning calorimetry (DSC; Q600, TA Instruments) were used. The thermal conductivity of the Ti-Nb-Cr-V-Ni was measured under an argon atmosphere increasing temperature from room temperature to 1000 °C at the rate of 50 °C/min. The specific heat was measured under the same conditions as the thermal conductivity measurement environment while increasing the temperature at a rate of 10 °C/min. Figure 9 presents the thermal conductivity and specific heat of Ti-Nb-Cr-V-Ni according to temperature. The thermal conductivity and specific heat of Ti-Nb-Cr-V-Ni increased with increasing temperature. The thermal conductivity was as low as 3.62 W/m·°C at room temperature and did not exceed 10 W/m·°C at 450 °C.

## 4. Analysis of LAM Processing

**Figure 10:**
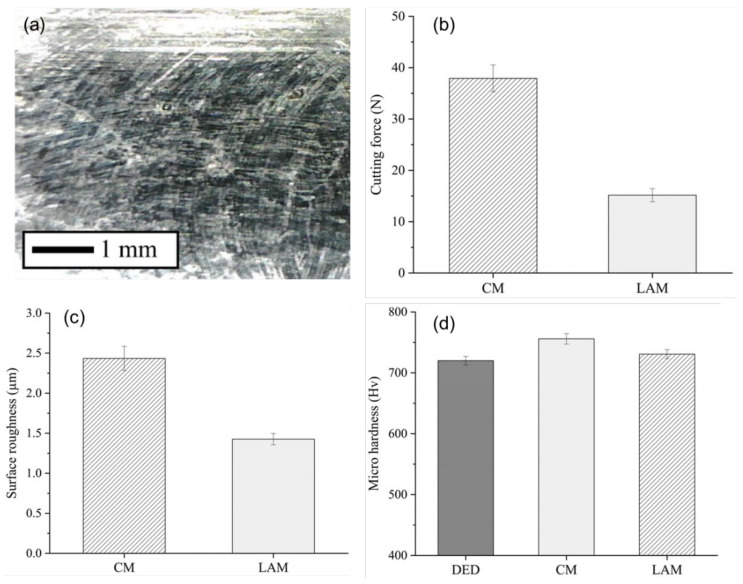
(**a**) machining surface after LAM, (**b**) the cutting forces, (**c**) surface roughness, and (**d**) microhardness of Ti-Nb-Cr-V-Ni.

For the cutting force, the feed cutting force ($F_{x}$) and the radial cutting force ($F_{y}$) were measured in three times repeated experiments, and the average value of the resultant force ($F_{c}$) was used. Figure 10a shows the SEM image for LAM. Figure 10b shows the cutting forces of Ti-Nb-Cr-V-Ni after post-processing. LAM reduced cutting forces by 60.0% compared to CM. The reduction in the cutting force is analyzed as the machining load has decreased due to softening for the laser preheating. The Ti-Nb-Cr-V-Ni exhibits brittle properties at low temperatures and high strength. The strength of a material is proportional to its cutting force. However, at high temperatures above 800 °C, Ti-Nb-Cr-V-Ni softens. When machining a workpiece is softened by laser preheating, the load, vibration, and resistance of machining are reduced, and LAM exhibited decreased cutting force compared with CM.

The surface roughness of Ti-Nb-Cr-V-Ni was measured using the surface profilometry equipment (Mitutoyo, SJ-201, Kawasaki, Japan) with 500 gf as the centerline average height ($R_{a}$) Of the cutting depth direction. $R_{a}$ which defines the arithmetic average of the profile height deviations from the mean line as given in Equation (1):

(1) $$R_{a}=\frac{1}{l}\int_{0}^{l}\left|z\left(x\right)dx\right|$$

where l is the evaluation length, and $z_{x}$ is the distance for the measurement point on the surface profile from the mean line [29,30]. For $R_{a}$, the roughness of the five-area randomly machining surfaces was measured three times and the average value was used. Figure 10c shows surface roughness of Ti-Nb-Cr-V-Ni after post-processing. The surface roughness of LAM was improved by 41.4% compared to CM. The Ti-Nb-Cr-V-Ni, which is brittle with high hardness and strength, delamination of metal pieces that were not removed in CM were delaminated during machining. Therefore, craters were formed on a part of the machined surface, but delaminating did not appear on the machined surface of LAM. This is analyzed as the processing machining has decreased due to softening for the laser preheating as depicted in Figure 10a.

The average microhardness of Ti-Nb-Cr-V-Ni was measured using the micro-Vickers hardness testing machines (Mitutoyo, HM-200) to be 720 HV before machining. Figure 10d shows the results of microhardness. The high microhardness was analyzed because of the refinement of grains due to rapid cooling, which is characteristic of the DED process. It was also analyzed that solid solution strengthening and phase stabilization rapidly according to high mixing configurational entropy were also influential. Microhardness after CM increased by 4.98%, and LAM increased by 1.48% compared to the state before machining. This increase can be analyzed as work hardening due to frictional heating and cutting forces occurring during machining. Notably, microhardness after LAM was slightly lower than after CM, as the laser preheating effect led to material softening, reducing the cutting force, and mitigating work hardening.

**Figure 11:**
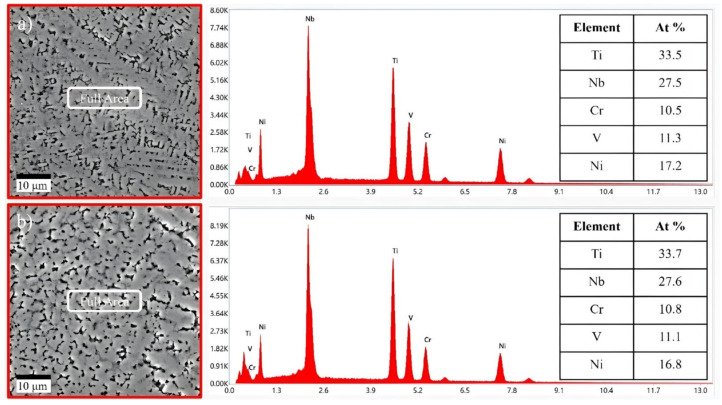
The results of EDS analysis for Ti-Nb-Cr-V-Ni after (**a**) CM and (**b**) LAM.

To further confirm the chemical composition of the LAM machined sample, EDS analysis was conducted for the machined surface. As a result of the analysis, the chemical composition of the machined surface remained unchanged even after post-processing with CM and LAM. Therefore, it is analyzed that LAM conducted as post-processing removes all the HAZ, and there is no change in chemical composition due to laser heating. Figure 11 shows the results of EDS analysis for Ti-Nb-Cr-V-Ni after post-processing.

**Figure 12:**
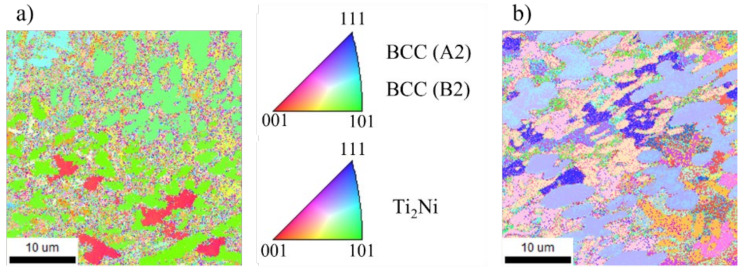
The results of EBSD analysis for Ti-Nb-Cr-V-Ni after (**a**) CM and (**b**) LAM.

The EBSD analysis was conducted to analyze changes in microstructure according to machining. The grain size after CM measured 2.20 μm and after LAM measured 3.18 μm both decreased from the grain size before machining, 3.66 μm. Figure 12 shows the results of EBSD analysis for Ti-Nb-Cr-V-Ni after post-processing. According to the Hall–Petch equation, that connection between grain size and the mechanical characteristics, the yield strength rises with a reduction in grain size. The Hall−Petch equation is expressed in Equation (2).

(2) $$\sigma_{y}=\sigma_{0}+\frac{k}{\sqrt{d}}$$

where $\sigma_{y}$ is the yield strength, $\sigma_{0}$ and k are the constant, and d is the grain size [31].

The decrease in grain size is analyzed to be due to work hardening. The heat generated by the machining load causes the grain to change, which in turn changes the mechanical properties of the material. Most of the HAZ generated by LAM were removed by machining, and work hardening hardly occurred due to the low machining load due to the softening of the material [22]. Conversely in CM, because of the high machining load, the mechanical properties were changed, leading to a reduction in grain size.

## 5. Analysis of Thermally Simulated Process

Analysis of the thermally simulated process was conducted to calculate the cutting depth used for LAM conditions. The changes in strength and properties that occur in preheated materials are critical in LAM. Specifically, the strength of Ti-Nb-Cr-V-Ni was observed to decrease rapidly while ductility increased within the temperature range of 800 °C to 1000 °C. A significant phase transition occurred when the temperature exceeded 980 °C. The primary objective of LAM is to machine the heat-affected zone (HAZ), which alters material properties during the softening that occurs at recovery and recrystallization temperatures. LAM operates with a low machining load, effectively softening materials and removing the HAZ to ensure no adverse changes in material properties. Consequently, the optimal preheating temperature for the workpiece was determined to be 980 °C, where strength reduction is most pronounced.

The governing equation of the thermally simulated process is as follows:

(3) $$\frac{k}{\rho C}\left(\frac{\partial^{2}T}{\partial x^{2}}+\frac{\partial^{2}T}{\partial y^{2}}+\frac{\partial^{2}T}{\partial z^{2}}\right)+\dot{Q\;}=\frac{\partial T}{\partial t}$$

where k is the thermal conductivity, ρ is the density, C is the specific heat, T is the temperature, $\dot{Q}$ is the power generation per unit volume and t is the time [32]. The initial condition (*t* = 0) is defined as follows:

(4) $$T\left(x,y,z,0\right)=T_{0}$$

**Figure 13:**
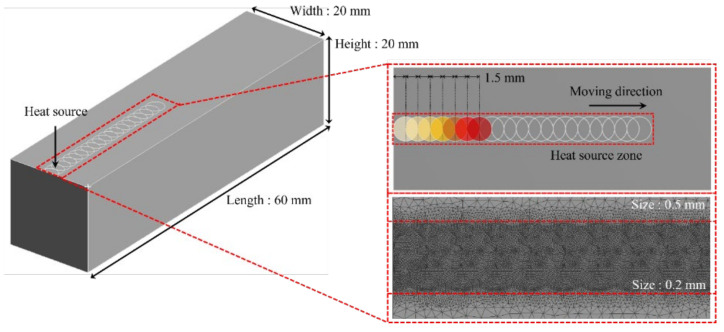
The 3D simulation model and concept of moving heat source.

The boundary condition is defined as follows:

(5) $$q\left(x,y\right)-h\left(T-T_{0}\right)=-k\frac{\partial T}{\partial z}\;$$

where q is the heat flux, h is the heat transfer coefficient, T is the surface temperature, and $T_{0}$ is the ambient temperature [32]. Analysis of the thermally simulated process was conducted through transient thermal analysis using ANSYS workbench. Moving heat source analysis for laser heat source derives temperature distribution by applying the temperature to the laser position according to time to the laser modeled at 1.5 mm intervals [33]. Figure 13 shows the simulation model for analysis of the thermally simulated process and the schematic of the moving heat source.

**Figure 14:**
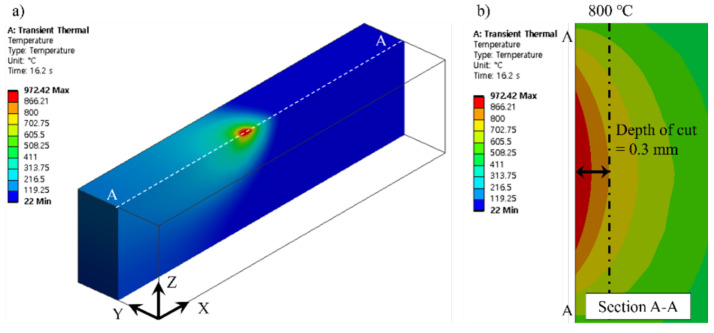
The results of the result of the thermally simulated process (**a**) temperature distribution of the workpiece and (**b**) cross-sectional view.

The simulation model was constructed of the tetrahedron mesh for 830,794 elements and 1,222,335 nodes. The heat source zone mesh size was applied to 0.2 mm and all other faces mesh sizes except for the heat source zone were applied to 0.5 mm. The laser beam diameter was 3 mm, and the feed rate was 100 mm/min. In the boundary conditions, k, ρ, and C were applied as measured values of Ti-Nb-Cr-V-Ni. The q was applied to 200 W as laser power, and h was applied to 5 W/m^2^-k as natural convection. Figure 14 shows the result of the thermally simulated process for laser preheating of Ti-Nb-Cr-V-Ni.

The results of the thermally simulated process indicated that the surface temperature of Ti-Nb-Cr-V-Ni reached approximately 930 °C, while the temperature at a depth of 0.3 mm was around 800 °C. Therefore, the optimal preheating temperature for Ti-Nb-Cr-V-Ni was confirmed to be 800 °C, establishing an efficient cutting depth of 0.3 mm. These findings highlight the importance of precise thermal management in enhancing machining efficiency and maintaining material integrity during LAM processes.

## 6. Conclusions

This research concentrated on fabricating a refractory Ti-Nb-Cr-V-Ni high-entropy alloy via Directed Energy Deposition (DED). For post-processing, the machining properties and efficiency were assessed using Conventional Machining (CM) and Laser-Assisted Machining (LAM). The following are the main findings:

1. The deposited Ti-Nb-Cr-V-Ni alloy showed BCC A2 and BCC B2 phases and a precipitated Ti_2_Ni phase.
2. The alloy indicated improved tensile strength and brittleness, with significant softening occurring above 800 °C, significantly reducing tensile strength. In addition, the thermal conductivity and specific heat rose with temperature, whereas tensile strength also dropped.
3. According to thermally simulated processes, the ideal preheating temperature for LAM was 800 °C, and the cutting depth was set at 0.3 mm.
4. Microhardness
5. Increased by 1.48% for LAM and 4.98% for CM, corresponding to a reduction in grain size from 3.66 μm to 2.20 μm following CM and 3.18 μm after LAM. This decrease is associated with work hardening due to machining heat.
6. In LAM, machining eliminated the majority of heat-affected zones (HAZ), leading to negligible work hardening due to a 60.0% decrease in cutting force relative to conventional machining (CM) that elevated loads induced more significant changes in mechanical properties and additional grain size reduction.
7. LAM’s surface roughness improved by 41.4% compared to CM, but the chemical composition of Ti-Nb-Cr-V-Ni remained unaffected following post-processing with both.

These findings demonstrate that post-processing with LAM is essential for developing the properties of refractory high-entropy alloys like Ti-Nb-Cr-V-Ni and unlocking their potential for advanced manufacturing applications.

## Figures and Tables

**Table 1:** The alloy composition for Ti-Nb-Cr-V-Ni.

Element (at%)	Ti	Nb	Cr	V
	33.0	28.0	11.0	11.0

**Table 2:** The processing conditions of DED and CM, LAM.

Parameter	Value	Parameter	Value
Laser power (W)	2000	Machining method	CM, LAM
Laser scanning speed (mm/s)	10	Preheating temperature (°C)	930
Powder feed rate (g/min)	13	Feed rate (mm/min)	100
Hatch spacing (mm)	1.34	Depth of cut (mm)	0.3
Shield gas flow rate (L/min)	15	Spindle speed (rpm)	10,000

**Table 3:** The alloy compositions for structure constituents of Ti-Nb-Cr-V-Ni.

Element, at%	Ti	Nb	Cr	V	Ni
BCC A2 phase	34.5	27.1	9.2	10.9	18.3
BCC B2 phase	31.8	22.0	8.3	11.6	26.3
Ti_2_Ni phase	31.1	22.2	9.3	10.6	26.8
Ti-Nb-Cr-V-Ni area	34.1	26.2	10.2	12.8	16.7
Designed composition	33	28	11	11	17

## Data Availability

This article is part of an ongoing research. Data are available upon request to the corresponding author.
